# Recovery from the DNA Replication Checkpoint

**DOI:** 10.3390/genes7110094

**Published:** 2016-10-28

**Authors:** Indrajit Chaudhury, Deanna M. Koepp

**Affiliations:** Department of Genetics, Cell Biology and Development, University of Minnesota, Minneapolis, MN 55455, USA; chaud072@umn.edu

**Keywords:** DNA replication, S-phase checkpoint, checkpoint recovery, fork restart

## Abstract

Checkpoint recovery is integral to a successful checkpoint response. Checkpoint pathways monitor progress during cell division so that in the event of an error, the checkpoint is activated to block the cell cycle and activate repair pathways. Intrinsic to this process is that once repair has been achieved, the checkpoint signaling pathway is inactivated and cell cycle progression resumes. We use the term “checkpoint recovery” to describe the pathways responsible for the inactivation of checkpoint signaling and cell cycle re-entry after the initial stress has been alleviated. The DNA replication or S-phase checkpoint monitors the integrity of DNA synthesis. When replication stress is encountered, replication forks are stalled, and the checkpoint signaling pathway is activated. Central to recovery from the S-phase checkpoint is the restart of stalled replication forks. If checkpoint recovery fails, stalled forks may become unstable and lead to DNA breaks or unusual DNA structures that are difficult to resolve, causing genomic instability. Alternatively, if cell cycle resumption mechanisms become uncoupled from checkpoint inactivation, cells with under-replicated DNA might proceed through the cell cycle, also diminishing genomic stability. In this review, we discuss the molecular mechanisms that contribute to inactivation of the S-phase checkpoint signaling pathway and the restart of replication forks during recovery from replication stress.

## 1. Introduction

The DNA replication or S-phase checkpoint monitors the integrity of DNA synthesis. Perturbations in DNA synthesis—such as a scarcity of free nucleotides or damaged DNA—leads to replication fork stalling and activation of the checkpoint pathway [1,2]. The replication checkpoint promotes cell viability by mediating a transcriptional response [3], stabilizing replication forks [1,2,4,5], suppressing firing of origins of replication [6,7], and stalling DNA synthesis [6,8]. Difficult to replicate DNA regions, such as repetitive sequences or fragile sites, can also stall forks and lead to activation of the checkpoint. Thus, even an S-phase in ideal environmental conditions can lead to multiple activations of the checkpoint, although these activations may be local rather than global.

Recovery from checkpoint activation is key to a successful checkpoint mechanism. During checkpoint recovery, the checkpoint signaling pathway is inactivated, and cell cycle progression is resumed. As the S-phase checkpoint is sensitive to perturbations even under favorable conditions, it is likely that recovery from checkpoint initiation is critical in each and every cell division. It is clear that failure to activate the S-phase checkpoint has deleterious consequences. Stalled forks may become unstable and lead to DNA breaks or unusual DNA structures that are difficult to resolve, leading to genomic instability. Similarly, if checkpoint recovery mechanisms fail, stalled forks can persist, increasing the likelihood of DNA damage. Alternatively, if cell cycle resumption mechanisms become uncoupled from checkpoint inactivation, cells with under-replicated DNA would proceed through the cell cycle, also impacting genomic stability.

## 2. Activation of Checkpoint Signaling

Initiation of the S-phase checkpoint response is dependent on a signaling cascade that is remarkably conserved in eukaryotes. This review will highlight mechanisms identified in simple eukaryotes such as budding yeast and point out distinctions observed in higher eukaryotes, including humans. Both stalled replication forks and DNA damage are recognized by sensor complexes, which activate a kinase cascade to prevent cell cycle progression (for review, see [9,10]). In budding yeast, sensing of DNA damage or stalled replication forksSeSeSasdfasdfasieuyghwq relies on the Rad24-dependent loading of the heterotrimeric Rad17-Mec3-Ddc1 (9-1-1 complex in fission yeast and humans) sliding clamp onto DNA [11,12,13]. This leads to Mec1 kinase (ATR in humans) activation, followed by the downstream phosphorylation and activation of the primary signaling kinase Rad53 [14,15]. In higher eukaryotes, ATR activation primarily leads to Chk1 kinase activation during the S-phase checkpoint, rather than Rad53 homolog Chk2 [16,17,18,19]. Mec1-dependent activation of Rad53 requires the adaptor Mrc1 (Claspin in humans), which forms a complex to stabilize replication forks at sites of replication stress [1,2,4,5]. Several other proteins function to promote Rad53 activation, including Rad9, Csm3, and Tof1 [4,20,21]. Csm3 and Tof1 form a complex with Mrc1 at replication forks [22], whereas Rad9 typically functions during the DNA damage checkpoint response, but it can substitute for Mrc1 under specific conditions [4,21]. Sgs1 helicase—a member of the RecQ helicase family and yeast ortholog of the human Bloom Syndrome protein BLM—is important for recruiting Rad53 to stalled forks and in maintaining association of DNA polymerases α and ε with the replication fork during checkpoint activation [23,24,25]. Mrc1 appears to also have a role during DNA replication in the absence of replication stress. Mrc1 is loaded onto replication origins and travels with the replisome complex at the replication fork [5,26,27].

While substantial progress has been made in identifying factors, pathways, and molecular events central to checkpoint activation, our understanding of checkpoint recovery is much more limited in comparison. Recovery from replication stress occurs after the original damage or defect is repaired, thus triggering checkpoint inactivation and a return to progression through the cell cycle [28,29].

## 3. Checkpoint Signaling Inactivation

Checkpoint initiation programs must be counteracted to achieve recovery and re-entry into the cell cycle. S-phase checkpoint recovery has to accomplish two key steps: inactivation of checkpoint signaling and resumption of DNA replication.

**Figure 1:**
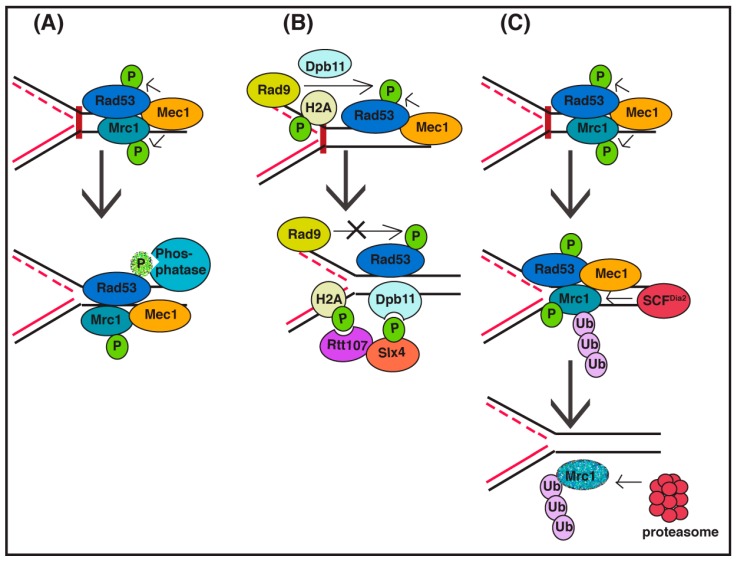
Diagram of DNA replication checkpoint signaling inactivation mechanisms. (**A**) During recovery, phosphatases from the PP2A and PP2C families de-phosphorylate Rad53, thus abrogating checkpoint signaling; (**B**) Competition for binding Rad9 (a Rad53 adaptor) by the Rtt107/Slx4 complex can dampen checkpoint signaling; (**C**) Ubiquitin-mediated degradation of the Rad53 kinase adaptor Mrc1, facilitated by the SCF^Dia2^ ubiquitin ligase, promotes checkpoint recovery.

One straightforward way to turn off a signaling pathway is by inhibiting key enzymatic steps in the pathway. There are S-phase checkpoint recovery mechanisms that involve deactivation of the checkpoint signaling pathway by interfering with Rad53 kinase activity (Figure 1A). Direct inactivation of the Rad53 signaling kinase by the action of PP2A-like phosphatase complex (Pph3/Psy2) has been observed to be important in S-phase checkpoint recovery [30,31,32,33]. Disruption of the Rad53 phosphatase complex leads to a defect in replication fork restart [34], suggesting that fork restart mechanisms are dependent on the inactivation of checkpoint signaling. Inhibition of a checkpoint kinase is also observed in the DNA damage checkpoint pathway, as PP2C-type phosphatases inhibit Rad53 signaling in this pathway [30,35]. As the initial checkpoint kinase activated during the S-phase checkpoint differs from the kinase activated during the DNA damage checkpoint, it follows that distinct Rad53 residues may need to be de-phosphorylated in each case [33]. Thus, the initiating event that triggers a specific checkpoint signaling pathway may determine (in part) the mechanism of inactivation during recovery.

Checkpoint signaling may also be affected by alterations of chromatin structure and changes to checkpoint protein complex architecture (Figure 1B). Dampening of checkpoint signaling can also be accomplished by Slx4-Rtt107 competition for Rad9 binding at sites of DNA lesions [36]. Slx4 and Rtt107 are scaffold proteins that can be recruited to stressed replication forks [37] as well as double-strand DNA breaks, uncapped telomeres, and other DNA lesions to displace Rad9 by competing for checkpoint-induced phosphosites on histone H2A, thus reducing the activation of Rad53 kinase and the checkpoint signaling pathway [38,39]. Indeed, evidence indicates that local action of the Slx4/Rtt107 complex at replication forks is complementary to the global activity of the Pph3/Psy2 Rad53 phosphatase [40]. In addition, chromatin remodeling factors Ino80 and Isw2—demonstrated to promote chromatin accessibility—attenuate S-phase checkpoint signaling and promote the recovery of stalled replication forks, although the mechanism by which this is accomplished in not known [41,42].

Protein degradation of checkpoint adaptor proteins is also important for S-phase checkpoint recovery, although whether this degradation is required solely for checkpoint signaling inactivation is not clear. Degradation of human Claspin (an adaptor for the Chk1 signaling kinase) is linked to checkpoint recovery after fork stalling caused by exposure to hydroxyurea, which limits free nucleotides [43,44,45]. Plk1-induced phosphorylation of Claspin triggers its degradation, facilitated by the SCFβ^TrCP^ ubiquitin ligase and the proteasome [43,44,46]. Importantly, degradation of Claspin also reduces Chk1 kinase signaling, thus inhibiting checkpoint signaling. This pathway is highly regulated, as a number of de-ubiquitinating enzymes have been identified that act to stabilize the Claspin protein, including USP7, USP28, USP29, and HERC2/USP20 [47,48,49,50,51]. Other types of replication stress also trigger Claspin degradation, but the ubiquitination pathway may differ, as the BRCA1 ubiquitin ligase can target Claspin degradation in response to topoisomerase inhibition [52].

In budding yeast, the Claspin ortholog Mrc1 is also targeted for degradation during recovery from the S-phase checkpoint (Figure 1C), indicating that removal of Mrc1 function during recovery is evolutionarily conserved. In this case, Mrc1 has been shown to be targeted for degradation via the SCF^Dia2^ ubiquitin ligase during recovery from the DNA alkylating agent methyl methanesulfonate (MMS). Both checkpoint-phosphorylated Mrc1 and unmodified Mrc1 are degraded, and a Mrc1 mutant protein that cannot be phosphorylated by checkpoint kinases exhibits partial stabilization. Induced degradation of Mrc1 only during recovery rescues the checkpoint recovery defect in cells lacking SCF^Dia2^ ubiquitin ligase activity, indicating that the predominant role of this complex during S-phase checkpoint recovery is degradation of Mrc1. However, induced degradation of a checkpoint-defective version of Mrc1 during the same time period cannot rescue the recovery defect in these cells, suggesting that removal of checkpoint-activated Mrc1 is key to the recovery process [53]. In addition, the Rtt101^Mms22^ ubiquitin ligase counteracts the replicative function of Mrc1 (although not via a degradation mechanism) to also facilitate replication fork restart or repair [54].

## 4. Resumption of DNA Replication

Completion of DNA replication after the activation of checkpoint signaling is critical to successful S-phase checkpoint recovery. During checkpoint activation, some proteins at the fork involved in checkpoint signaling, such as ATR/Mec1 and Claspin/Mrc1, promote replication fork stabilization [23,27,55,56,57], presumably to maintain forks so that they can be restarted after the replication stress is removed. Evidence suggests that in response to low deoxynucleotide triphosphates (dNTP) levels, Mec1 and Rad53 regulate replisome function rather than the integrity of the complex, as the replisome is stably associated with replication forks in the absence of Mec1 and Rad53 [58]. Additional proteins are recruited to help stabilize forks, many of which are also involved in fork restart mechanisms. Prolonged fork stalling may lead to the replisome moving away from the fork or replisome components dissociating from chromatin. Stalled forks may also undergo structural rearrangements such as fork reversal and rewinding of parental and newly-replicated DNA strands into “chicken foot” structures that are difficult to resolve.

A growing number of proteins have been identified to have roles in replication fork restart, and multiple fork restart mechanisms have been proposed. In general, these mechanisms can be divided into two groups: direct restart or a broad group of alternative restart mechanisms that require remodeling or recombination to restore DNA replication. The type of replication stress or DNA lesion that led to the fork stall may influence the type of restart mechanism. For example, recovery of a fork stalled by limiting replication components presents a different challenge than a leading strand lesion that has a led to uncoupling of the leading and lagging strand polymerases.

Direct restart of a stable, stalled fork is a straightforward approach to complete DNA synthesis during checkpoint recovery. However, we know very little about mechanistic steps required for initiation of direct restart of a stalled fork (re-priming) in eukaryotic cells. For example, it is not clear if re-priming is linked to inhibition of checkpoint signaling. In primates, the methyltransferase and nuclease protein METNASE (SETMAR) is required for restart of the majority of forks following a hydroxyurea-induced checkpoint [59,60]. Interestingly, METNASE is involved in a feedback mechanism with Chk1, in which Chk1-mediated phosphorylation of METNASE decreases its function in fork restart and increases Chk1 protein stability [61,62], thereby prolonging checkpoint activation and preventing premature fork restart. Thus, substantial coordination between checkpoint signaling inactivation and fork restart mechanisms may exist.

Alternative ways to restart DNA replication include a variety of mechanisms that may require remodeling by fork reversal, nucleolytic processing of nascent DNA strands, or recombination mechanisms (for comprehensive review, see [63,64]). Proteins capable of fork reversal include the Rad5 helicase in yeast [65] (and human ortholog HLTF [66]) and Fanconi Anemia protein FANCM [67] and its budding yeast homolog Mph1 [68], as well as its fission yeast homolog Fml1 [69]. In higher eukaryotes, fork reversal in vivo also depends on poly (ADP-ribose) polymerase (PARP1) [70]. In humans, the helicase SMARCAL1 can remodel forks to achieve branch migration and trigger fork restart [71,72,73,74,75]. The EEPD1 nuclease is recruited to stalled forks, and promotes DNA end resection and fork restart [76].

Recombination-based restart mechanisms are probably most relevant to collapsed forks where the replication machinery has been lost, thus facilitating Holliday junction formation. The recombination factor Rad51 (which catalyzes Holliday junctions) can be recruited to stalled forks [77,78,79,80]. Helicases that function in Holliday junction resolution during recombination, including the RecQ helicase family members Bloom Syndrome protein BLM and the Werner Syndrome protein WRN have demonstrated roles in fork restart [81,82,83]. This activity is conserved, as the related protein in budding yeast, Sgs1, is important for recombination-mediated fork restart [84,85]. Replication fork restart is also linked to Fanconi Anemia (for review, see [86]). Although members of this group are best known for their roles in interstrand crosslink repair, the FANCD1, FANCD2, and FANCJ proteins have distinct roles in replication fork restart [87]. In particular, FANCD2 is required to stabilize and recruit BLM to stalled forks [88]. In addition to recombination factors, conserved scaffold proteins such as Slx4 and Rtt107 that interact with structure-specific nucleases or fork repair proteins are also important for fork restart [89,90,91,92,93,94,95]. Finally, forks that cannot be recovered may be bypassed by the firing of nearby “back-up” origins, ensuring that chromosome duplication is completed [96,97].

## 5. Future Perspectives

We are just beginning to identify and investigate mechanisms of checkpoint signal inactivation. As these mechanisms become better understood, it will be interesting to determine whether they are coordinated into an overall cellular program that facilitates recovery from the S-phase checkpoint. One intriguing question is whether checkpoint activation in response to distinct damage or replication stress triggers specific signaling inactivation mechanisms. Moreover, are checkpoint recovery mechanisms themselves downstream targets of the initial checkpoint activation? It is tempting to imagine that mechanisms that turn off the signaling pathway are folded into the initial activation as a means of limiting prolonged activation.

Many of the proteins required for the DNA replication checkpoint and fork restart during recovery are compromised in human diseases. It is easy to imagine that defects in checkpoint recovery might lead to genome instability, and therefore also contribute to human disease. As checkpoint recovery mechanisms become better understood, we look forward to new information about the role of these pathways in protecting human health.

## Abbreviations and Protein Name Derivations

Rad	Radiation sensitive
Mec	Mitosis entry checkpoint
Ddc1	DNA damage checkpoint 1
ATR	ataxia telangiectasia mutated- and Rad3-related
Chk	Checkpoint kinase
Mrc1	Mediator of replication checkpoint protein 1
Csm3	Chromosome segregation in meiosis protein 3
Tof1	Topoisomerase 1-associated factor 1
Sgs1	slow growth suppressor 1
RecQ	recombination Q family
BLM	Bloom Syndrome protein
PP2A	protein phosphatase 2A
Pph3	protein phosphatase 3
Psy2	platinum sensitivity 2
PP2C	protein phosphatase 2C
Slx4	Structure-Specific Endonuclease Subunit
Rtt	Regulator of Ty1 transposition protein
Ino80	inositol requiring 80
Isw2	imitation switch 2
Plk1	Polo-like kinase 1
SCF	Skp, Cullin, F-box protein containing complex
βTrCP	beta-transducin repeat protein
USP	ubiquitin-specific protease
HERC2	HECT And RLD Domain Containing E3 Ubiquitin Protein Ligase 2
BRCA1	Breast cancer type 1 susceptibility protein
Dia2	Digs into agar 2
Mms22	Methyl Methanesulfonate sensitivity 22
METNASE	methyl transferase and nuclease
SETMAR	SET domain and mariner transposase fusion
HLTF	Helicase-like transcription factor
FANC	Fanconi Anemia group protein
Mph1	mutator phenotype 1
Fml1	FANCM ortholog
SMARCAL	SWI/SNF Related, Matrix Associated, Actin Dependent Regulator Of Chromatin, Subfamily A-Like 1
EEPD1	Endonuclease/exonuclease/phosphatase family domain-containing protein 1
WRN	Werner Syndrome protein
